# Blood Biomarkers as Prognostic Indicators for Neurological Injury in COVID-19 Patients: A Systematic Review and Meta-Analysis

**DOI:** 10.3390/ijms242115738

**Published:** 2023-10-30

**Authors:** Zhiwei Huang, Kassahun Haile, Lealem Gedefaw, Benson Wui-Man Lau, Ling Jin, Shea Ping Yip, Chien-Ling Huang

**Affiliations:** 1 Department of Health Technology and Informatics, The Hong Kong Polytechnic University, Hong Kong, China; wayne.huang@polyu.edu.hk (Z.H.); lealem.bimerew@connect.polyu.hk (L.G.); ling.jin@polyu.edu.hk (L.J.); 2 Department of Medical Laboratory Science, Wolkite University, Wolkite P.O. Box 07, Ethiopia; kassahaile07@gmail.com; 3 Department of Rehabilitation Sciences, The Hong Kong Polytechnic University, Hong Kong, China; benson.lau@polyu.edu.hk; 4 Department of Civil and Environmental Engineering, The Hong Kong Polytechnic University, Hong Kong, China

**Keywords:** glial fibrillary acidic protein, neurofilament light chain, meta-analysis, neurological biomarker, Coronavirus disease 2019

## Abstract

Coronavirus disease 2019 (COVID-19) has been linked to various neurological complications. This meta-analysis assessed the relationship between glial fibrillary acidic protein (GFAP) and neurofilament light chain (NfL) levels in the blood and neurological injury in COVID-19 patients. A comprehensive search of various databases was conducted until 18 August 2023, to find studies reporting GFAP and NfL blood levels in COVID-19 patients with neurological complications. GFAP and NfL levels were estimated between COVID-19 patients and healthy controls, and meta-analyses were performed using RevMan 5.4 software for analysis. In the 21 collected studies, it was found that COVID-19 patients had significantly higher levels of pooled GFAP (SMD = 0.52; 95% CI: 0.31, 0.73; *p* ≤ 0.001) and NfL (SMD = 0.60; 95% CI: 0.37, 0.82; *p* ≤ 0.001) when compared to the healthy controls. The pooled GFAP (SMD = 0.86; 95% CI: 0.26, 1.45; *p* ≤ 0.01) and NfL (SMD = 0.87; 95% CI: 0.48, 1.26; *p* ≤ 0.001) were significantly higher in non-survivors. These findings indicate a significant association between COVID-19 severity and elevated levels of GFAP and NfL, suggesting that GFAP and NfL could serve as potential diagnostic and prognostic markers for the early detection and monitoring of COVID-19-related neurological injuries.

## 1. Introduction

Coronavirus disease 2019 (COVID-19) is a respiratory illness caused by severe acute respiratory syndrome coronavirus-2 (SARS-CoV-2) [1]. While primarily affecting the respiratory system, COVID-19 has been associated with a range of neurological diseases [2,3], posing significant public health concerns. Common neurological manifestations include fatigue, headache, vision impairment, neuropsychiatric symptoms, encephalopathy, peripheral neuropathy, stroke, seizures, and cerebrovascular disease [4,5,6]. These symptoms can affect both adults and children, and their severity can vary widely [2,7].

Given the broad range of neurological symptoms associated with COVID-19, a reliable neurological biomarker is needed to detect and monitor neurological injuries in patients with the disease [8]. Blood biomarkers, such as glial fibrillary acidic protein (GFAP) and neurofilament light chain (NfL), have been identified as potential tools for detecting and monitoring central nervous system (CNS) injury. These biomarkers can directly detect the inflammatory response of invading pathogens or hosts [9,10]. On the other hand, these two biomarkers were initially applied to some neurodegenerative diseases such as Alzheimer’s disease (AD). For instance, Elaihi et al. observed elevated plasma levels of GFAP and NfL during the early stages of AD, with higher levels of these biomarkers being associated with more severe functional impairment [11]. Similarly, Baiardi et al. reported that GFAP and NfL could be employed to distinguish between different neurodegenerative diseases [12]. In recent years, a growing body of evidence suggests a connection between the pathogenesis of neurodegenerative diseases and immunological mechanisms. Studies have identified pathological astrocytosis around Aβ plaques in AD patients, leading to astrocytosis and increased expression of GFAP [13]. Furthermore, elevated NfL levels in asymptomatic AD gene carriers have been linked to cognitive scores, brain volume, ventricle size, hippocampus size, and longitudinal brain changes. This indicates the potential of NfL to reflect the clinical features of AD and aid in preclinical diagnosis [14].

GFAP is an intermediate filament highly expressed in astrocytes and has been suggested as a potential blood biomarker for astrocytic injury as well as participating in the pathophysiological functions of astrocytes, which are associated with nerve injury [15,16]. Studies have shown glial activation and neuronal injury in patients with COVID-19 [17,18] with elevated biomarkers related to neuronal injury detected in the cerebrospinal fluid (CSF) and blood of COVID-19 patients [19]. The concentrations of NfL and GFAP were found to be elevated in 405 non-hospitalized COVID-19 patients [20], with plasma GFAP levels observed to be significantly two-fold higher in critically ill patients with COVID-19 when compared to healthy controls [17]. Serum GFAP showed a significant association with the severity of COVID-19 infection [21], and both NfL and GFAP predicted COVID-19-associated mortality in hospitalized patients [18]. Blood GFAP has been recently considered as a potential biomarker of CNS disorders and their severity. For instance, serum GFAP levels increased in patients with severe brain injury on admission and predicted neurological outcomes at six months [22], and they also correlated with the extent of brain damage and severity of the stroke in patients with ischemic stroke [15].

NfL protein is a cylindrical protein exclusively located in the neuronal axon and is a dynamic marker of active neuronal damage [10,23]. It is considered a potential blood biomarker of neuronal damage/injury because of its enrichment in axons and its release into the bloodstream in significant quantities following neuronal injury [24,25]. COVID-19 patients have been shown to experience neuro-axonal injury, which puts them at risk of long-term neurological sequelae [19]. Several studies have reported elevated serum and CSF biomarkers indicating neurologic injury in COVID-19 patients [18,21,26], with significantly higher plasma NfL levels observed in COVID-19 patients when compared to healthy controls [27]. Elevated NfL levels were associated with worse clinical outcomes in COVID-19 patients, with high concentrations reported in critically ill COVID-19 patients and enhancing the prediction of COVID-19 mortality [19,27,28].

Both GFAP and NfL, typically confined to the CNS, can have a significant impact on neurological health and injuries when they enter the bloodstream. Under specific conditions, such as nervous system injury or inflammation, damaged cell membranes can release GFAP or NfL into surrounding tissues or CSF. Moreover, disruptions in the blood–brain barrier (BBB) may allow GFAP and NfL to cross through CSF to circulation. In some cases of neurological diseases and inflammatory states, the rupture of cell membranes or cell death can result in the release of these two biomarkers [29].

Taken together, previous studies have reported changes in NfL and GFAP levels in COVID-19 patients [18,19,21,26,27]. However, some studies have reported contradictory findings [30,31,32], which warrant a comprehensive analysis of the relationship between neurological biomarkers (GFAP and NfL) and COVID-19. Therefore, this systematic review and meta-analysis aimed to determine the pooled standardized mean differences (SMD) of GFAP and NfL between COVID-19 patients and healthy controls, generating evidence for the association between neurological injury-related biomarkers and COVID-19 prognosis.

## 2. Materials and Methods

### 2.1. Study Design

The study design for this systematic review and meta-analysis followed the Preferred Reporting Items for Systematic Reviews and Meta-Analysis (PRISMA) guidelines [33], which is a widely recognized framework for conducting transparent and rigorous systematic reviews and meta-analyses. The protocol was registered in INPLASY (ID: INPLASY202390063). The main objective of this study was to compare the SMD of two biomarkers, NfL and GFAP, between COVID-19 patients and healthy controls on a global scale. The pooled SMD values were used to assess the potential differences in the biomarker levels between the two groups. The study design involved a comprehensive literature search of relevant databases, including PubMed, EMBASE, and Web of Science, to identify all eligible studies published up to the date of the search. The titles and abstracts of the identified studies were independently screened by two reviewers (Z.H. and K.H.), followed by full-text screening to identify studies included in the final analysis. Data were extracted from the included studies, and a meta-analysis was performed to obtain the pooled SMD using appropriate statistical methods. This study aimed to provide a comprehensive overview of the differences in biomarker levels between COVID-19 patients and healthy controls globally, which may help identify potential diagnostic and prognostic markers for COVID-19.

### 2.2. Eligibility Criteria

This study included original articles with case–control, cohort, and cross-sectional study designs that measured human serum or plasma NfL and GFAP concentrations in both COVID-19 patients and healthy controls. The eligibility criteria also included studies that reported the outcome of interest (NfL and GFAP) and expressed the results as mean and standard deviation (SD) or median and interquartile range (IQR) for both COVID-19 patients and healthy controls. We included studies that were published from 9 July 2020 to 18 August 2023, regardless of their publication status, such as published, preprints, or grey literature. We excluded editorials, case reports, conference abstracts, non-full-text abstracts, systematic reviews and meta-analyses, expert opinions, animal studies, and studies not published in English. The primary outcome of this study was to determine the SMD of NfL and GFAP between COVID-19 patients and healthy controls. The inclusion and exclusion criteria were based on the PRISMA guidelines [33].

### 2.3. Search Strategies

To ensure our literature search was comprehensive, we systematically searched multiple electronic databases, including PubMed, Web of Science, Scopus, EMBASE, Google Scholar, and MedRxiv, from their inception until 18 August 2023. We used a combination of search terms to identify relevant studies: (“Coronavirus disease 2019” OR “Coronavirus 2019” OR “COVID-19” OR “COVID19” OR “Severe acute respiratory syndrome coronavirus 2” OR “SARS-CoV-2” OR “nCoV-2019” OR “2019-nCoV” OR “Novel coronavirus”) AND (“glial fibrillary acidic protein” OR “glial fibrillary acidic-protein” OR “glial fibrillary protein” OR “glial acidic protein” OR “GFAP” OR “sGFAP” OR “pGFAP” OR “sGFAP” OR “neurofilament light chain” OR “neurofilament-light chain” OR “neurofilament light chain protein” OR “neurofilament” OR “NfL” OR “sNfL” OR “pNfL” OR “neurological biomarker” OR “neurological injury related biomarker”).

To ensure relevant studies were not missed, we manually searched the reference lists of eligible studies in Google Scholar. The literature search was conducted independently by two authors (Z.H. and K.H.), and any discrepancies were resolved through discussion.

### 2.4. Data Collection Process

#### 2.4.1. Selection Process and Data Extraction

Two independent authors (Z.H. and K.H.) performed the selection process and data extraction, and any discrepancies were resolved through discussion until a consensus was reached. Extensive searching of electronic databases, including PubMed, Web of Science, Scopus, EMBASE, Google Scholar, and MedRxiv, was conducted to identify relevant studies. The results were imported into Microsoft Excel to remove duplicate articles and organize the data. Subsequently, the same two authors independently extracted the relevant information from the eligible studies by using a designed data extraction sheet in Microsoft Excel. The extracted information included the first author’s name, year of publication, study design, country, sample size, and severity of COVID-19. The mean and SD of GFAP and NfL levels were also extracted for both COVID-19 and healthy controls. In case the studies expressed the median and IQR of these biomarkers, they were extracted, and the data were converted into mean (µ) and SD with the formulae described previously (µ = q1 + m + q3/3 and SD = q3 − q1/1.35), where m: median; q1: interquartile 1; and q3: interquartile 3 [34]. The definition of COVID-19 severity was based on at least one of the following criteria: shortness of breath, admission to intensive care unit (ICU), a respiration rate of ≥30 times per minute, blood oxygen saturation of ≤93% at rest, or PaO_2_/FiO_2_ ratio of ≤300 mmHg [17,18,25,35,36,37]. PaO_2_/FiO_2_ ratio was defined as the ratio of arterial O_2_ partial pressure (PaO_2_ expressed in mmHg) to fractional inspired O_2_ (FiO_2_ expressed as a fraction) and is normally in the range of 400–500 mmHg. However, the definitions for COVID-19 severity varied among the included studies, which could have contributed to heterogeneity.

#### 2.4.2. Quality Assessment

The quality of all eligible studies was evaluated using the Newcastle–Ottawa scale (NOS) for case–control, cross-sectional, and cohort studies. This scale included the domains of participant selection, comparability, and outcome, with a total of nine stars assigned to all items. Studies with 5 or 6 NOS stars were considered moderate-quality studies, and those with seven or more stars were of high quality. The same two authors independently assessed the quality of the studies, and any discrepancies were resolved through discussion. If the authors could not resolve the discrepancies through discussion, they sought a third party, like a senior researcher, for consultation.

#### 2.4.3. Effect Measures

The primary analysis aimed to compute the SMD of GFAP and NfL and their 95% confidence intervals (CI) between COVID-19 patients and healthy controls through meta-analysis. The values of these neurological biomarkers in each group were extracted from the previous published studies’ reports.

### 2.5. Statistical Analysis

The statistical analysis was conducted using Review Manager version 5.4 (The Nordic Cochrane Centre, The Cochrane Collaboration, Copenhagen, Denmark). Tables and forest plots were used to summarize and present the results. The SMD value with 95% CI of GFAP and NfL was analyzed for COVID-19 and healthy controls. The random- and fixed-effects models were used for pooling the SMD analysis of GFAP and NfL between COVID-19 patients and healthy controls with their respective 95% CIs. The random-effects (RE) model was used when the I^2^ percentage was greater than 50%, while the fixed-effects model was used when the I^2^ percentage was lower than or equal to 50%. The I^2^ statistic was used to assess heterogeneity, and the funnel plot was used to evaluate publication bias. A *p* value ≤ 0.05 was considered statistically significant.

## 3. Results

### 3.1. Study Search, Selection, and Characteristics

**Figure 1:**
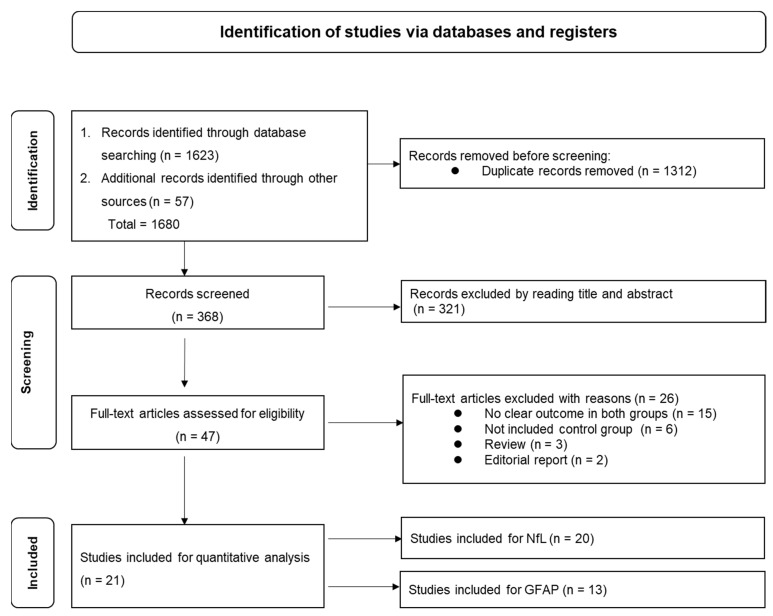
PRISMA workflow chart describes the selection of studies for the systematic review and meta-analysis on the relationship between GFAP and NfL with COVID-19. GFAP, glial fibrillary acidic protein; NfL, neurofilament light chain protein; n, number.

After a systematic search of the databases for studies published in English between 9 July 2020 and 18 August 2023, a total of 1680 records were identified from PubMed (*n* = 631), Web of Science (*n* = 313), Scopus (*n* = 340), EMBASE (*n* = 262), Google Scholar (*n* = 114), and MedRxiv (*n* = 20). After removing duplicates (*n* = 1312), 368 studies remained. Based on a review of the titles and abstracts, 321 studies were excluded. The full texts of the remaining 47 studies were reviewed, and 26 studies were subsequently excluded based on the eligibility criteria. Among the excluded studies, 15 did not report outcomes of interest (NfL and GFAP) in both COVID-19 and healthy controls [30,31,38,39,40,41,42,43,44,45,46,47,48,49,50], six did not include healthy controls [31,36,38,44,48,51], three were reviews [8,52,53], and two were editorial reports [54,55]; note that a few reports belonged to more than one category. Finally, 21 studies that reported mean (±SD) or median (±IQR) values for NfL and GFAP were included in the meta-analysis (Figure 1).

After careful screening, a total of 21 studies that met the inclusion and exclusion criteria were included in this systematic review and meta-analysis. Of these studies, four were conducted in Italy [18,25,27,56], three in Sweden [17,35,57], three in the UK [58,59,60], three in the USA [19,61,62], two in Turkey [27,37], two in Germany [32,63], two in Spain [26,64], one in Canada [65], and one in Norway [20]. 

For the meta-analysis of pooled GFAP, 13 studies with a total of 1896 participants (1413 COVID-19 patients and 483 healthy controls) were included (Table 1). Among 13 studies, there were five studies included in the meta-analysis of the relationship between GFAP and the severity of COVID-19 (mild, moderate, and severe) with 131, 188, and 246 patients with mild, moderate, and severe diseases, respectively. The remaining eight studies did not report GFAP values in patients with mild, moderate, and severe disease separately and were not included in the final meta-analysis. We conducted a risk of bias evaluation for each of the included studies and found that they had higher NOS scores, indicating a lower risk of bias.

In this systematic review and meta-analysis, a total of 20 studies were included in the pooled SMD analysis of NfL, comprising 5182 study participants (1978 COVID-19 patients and 3204 healthy controls) (Table 2). Among 20 studies, seven were included in the meta-analysis, which examined the association between NfL and the severity of COVID-19 in a total of 153, 208, and 222 patients with mild, moderate, and severe disease, respectively. The remaining 13 studies did not independently report NfL values according to the severity of patients and were not included in the final meta-analysis. We conducted a risk of bias evaluation for each of the included studies and found that they had higher NOS scores, indicating a lower risk of bias.

### 3.2. Meta-Analysis

#### 3.2.1. Elevated GFAP Level Is Associated with COVID-19

**Figure 2:**
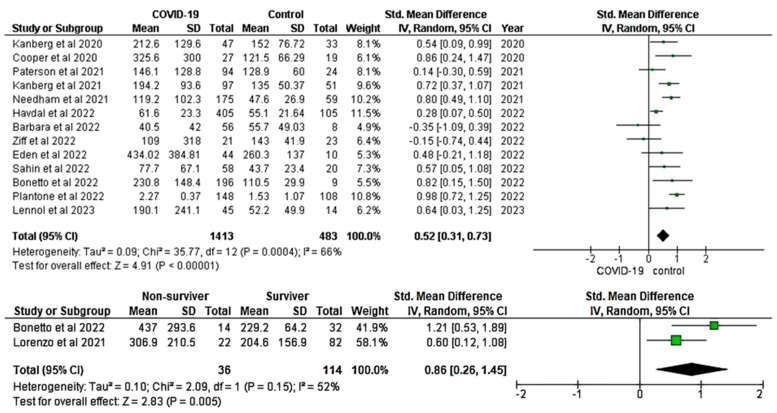
Pooled SMD of GFAP between COVID-19 patients and healthy controls [17,18,20,35,37,42,56,57,58,59,60,62,64,65]. Significantly increased pooled GFP was observed in COVID-19 patients (**top panel**) and non-survivors (**bottom panel**). A positive SMD value represents that the estimated value is significantly higher in COVID-19 patients than in healthy controls. The y-axis represents included studies; the x-axis represents the effect sizes of the estimated SMD. The square box (green) in the forest plot represents the SMD (effect estimate) of each study, and the area of the square box indicates the weight assigned to this particular study. The length of the horizontal line running across each study represents the width of the 95% CI for the SMD estimate for that particular study. The neutral point is plotted on the x-axis at the “0” mark. A vertical line passes through the neutral point, indicating the study groups (COVID-19 vs. Control). The black diamond represents the overall SMD estimate in the form of the inverse variance random-effects meta-analysis. CI, confidence interval; COVID-19, Coronavirus disease 2019; GFAP, glial fibrillary acidic protein; IV, inverse variance; SD, standard deviation; Std, standard.

Pooled SMDs were computed for 13 studies in the analysis of the association between GFAP levels in patients with COVID-19 and healthy controls, comprising a total of 1896 participants (1413 COVID-19 patients and 483 healthy controls). RE method was applied to calculate the pooled SMD of GFAP in the COVID-19 group when compared to the healthy controls with significant heterogeneity (I^2^: 66%; *p* ≤ 0.001). Under the REs model, the common effect size estimate of pooled SMD was 0.52 (95% CI: 0.31, 0.73). The overall pooled SMD value of GFAP indicated a significant increase in patients with COVID-19 when compared to the healthy controls (Z = 4.91; *p* ≤ 0.001) (top panel, Figure 2). Two studies were included in the meta-analysis that compared the levels of GFAP between COVID-19 survivor and non-survivor groups. Based on the RE model, the pooled SMD was 0.86 (95% CI: 0.26, 1.45; I^2^: 52%). The overall pooled SMD showed a significant difference between survivors and non-survivors of COVID-19 patients (Z = 2.83; *p* ≤ 0.01) (bottom panel, Figure 2).

#### 3.2.2. Increased GFAP Level Is Associated with COVID-19 Severity

**Figure 3:**
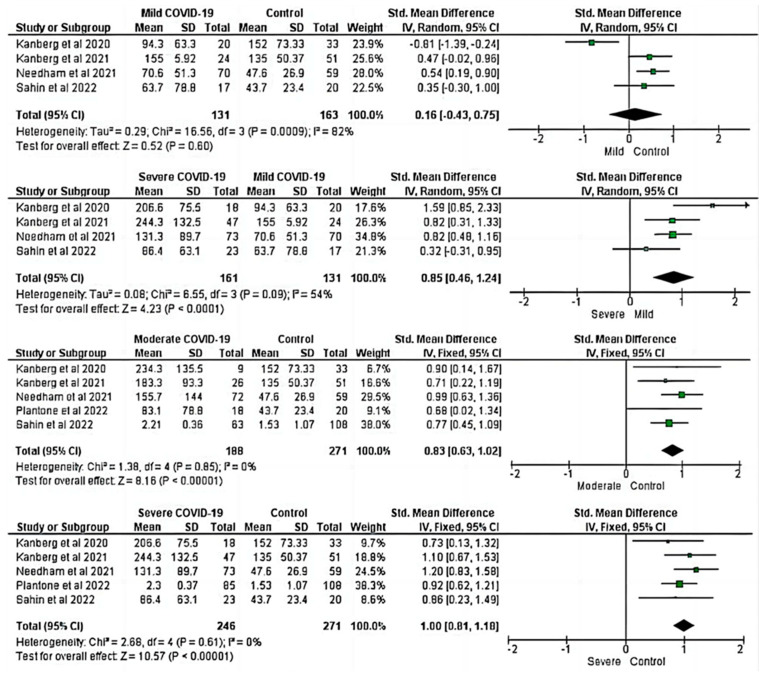
Forest plot analysis for the pooled SMD of GFAP between COVID-19 severity and healthy controls [17,18,35,37,59]. Increased blood GFAP level is associated with the severity of COVID-19. A positive SMD value represents that the estimated value is significantly higher in COVID-19 patients than in healthy controls. The y-axis represents included studies, and the x-axis represents the effect sizes of the estimated SMD. The square box (green) in the forest plot represents the SMD (effect estimate) of each study, and the area of the square box indicates the weight assigned to this particular study. The length of the horizontal line running across each study estimate represents the width of the 95% CI for the SMD estimate in the particular study. The neutral point is plotted on the x-axis at the “0” mark. A vertical line passes through the neutral point, indicating the study group (COVID-19 vs. Control). The black diamond represents the overall SMD estimate in the form of the inverse variance random- or fixed-effects meta-analysis. CI, confidence interval; COVID-19, Coronavirus disease 2019; IV, inverse variance; SD, standard deviation; Std, standard.

Four studies that reported GFAP levels in COVID-19 patients with mild disease and healthy controls were included in the comparison of GFAP levels between patients with mild COVID-19 and healthy controls. RE meta-analysis of the overall pooled GFAP revealed no significant differences between healthy controls and patients with mild COVID-19 (SMD = 0.16; 95% CI: −0.43, 0.75; Z = 0.52; *p* > 0.05) with heterogeneity (I^2^: 82%) (first panel from the top, Figure 3). However, the overall pooled GFAP level showed a significant difference between COVID-19 patients with severe and mild disease (SMD = 0.85; 95% CI: 0.46, 1.24; Z = 4.23; *p* ≤ 0.001) with heterogeneity (I^2^: 54%) (second panel from the top, Figure 3), indicating that the concentration of biomarkers in mild COVID-19 was lower than the severe cases. Furthermore, five studies were included in the meta-analysis of pooled GFAP levels between moderate and severe COVID-19 and healthy controls. The fixed-effects model showed that moderate COVID-19 patients had higher levels of pooled GFAP when compared to the healthy controls (SMD = 0.83; 95% CI: 0.63, 1.02; Z = 8.16; *p* ≤ 0.001; I^2^: 0%) (the third panel from the top, Figure 3). Fixed-effects meta-analysis of the overall pooled GFAP revealed a significant difference between patients with severe COVID-19 and healthy controls (SMD = 1.00; 95% CI: 0.81, 1.18; Z = 10.57; *p* ≤ 0.001; I^2^: 0.0%) (bottom panel, Figure 3). This implies that increased GFAP could indicate the severity of COVID-19-associated neurological damage [21,22].

#### 3.2.3. Elevated NfL Level Is Associated with COVID-19

**Figure 4:**
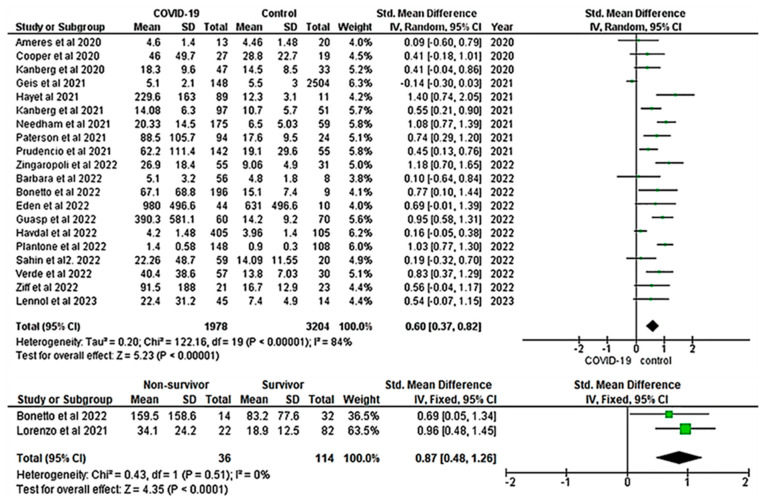
Forest plot for the pooled SMD analysis of NfL between COVID-19 and healthy controls [17,18,19,20,25,26,27,32,35,37,42,56,57,58,59,60,61,62,63,64,65]. Significantly increased pooled NfL was observed in COVID-19 patients (**top panel**) and non-survivors (**bottom panel**). A positive SMD value represents that the estimated value is significantly higher in COVID-19 patients than in healthy controls. The y-axis represents included studies, and the x-axis represents the effect sizes of the estimated SMD. The square box (green) in the forest plot represents the SMD (effect estimate) of each study, and the area of the square box indicates the weight assigned to this particular study. The length of the horizontal line running across each study estimate represents the width of the 95% CI for the SMD estimate in the particular study. The neutral point is plotted on the x-axis at the “0” mark. A vertical line passes through the neutral point, indicating the study group (COVID-19 vs. Control). The black diamond represents the overall SMD estimate in the form of the inverse variance random- or fixed-effects meta-analysis. CI, confidence interval; COVID-19, Coronavirus disease 2019; IV, inverse variance; SD, standard deviation; Std, standard.

Twenty studies comprising a total of 5182 participants (1978 COVID-19 patients and 3204 healthy controls) were included in the meta-analysis of the association between NfL levels and COVID-19. The RE meta-analysis showed that patients with COVID-19 had significantly higher levels of NfL when compared to the healthy controls (SMD = 0.60; 95% CI: 0.37, 0.82; Z = 5.23; *p* ≤ 0.001), with significant heterogeneity across studies (I^2^: 84%; *p* ≤ 0.001) (top panel, Figure 4)**.** To determine the relationship between NfL and COVID-19 mortality, we included two studies in our analysis. The overall pooled fixed-effects meta-analysis revealed a significant difference in the pooled value of NfL between survivors and non-survivors groups of COVID-19 (SMD = 0.87; 95% CI: 0.48, 1.26; Z = 4.35; *p* ≤ 0.001; I^2^: 0.0%) (bottom panel, Figure 4).

#### 3.2.4. Increased NfL Level Is Associated with COVID-19 Severity

**Figure 5:**
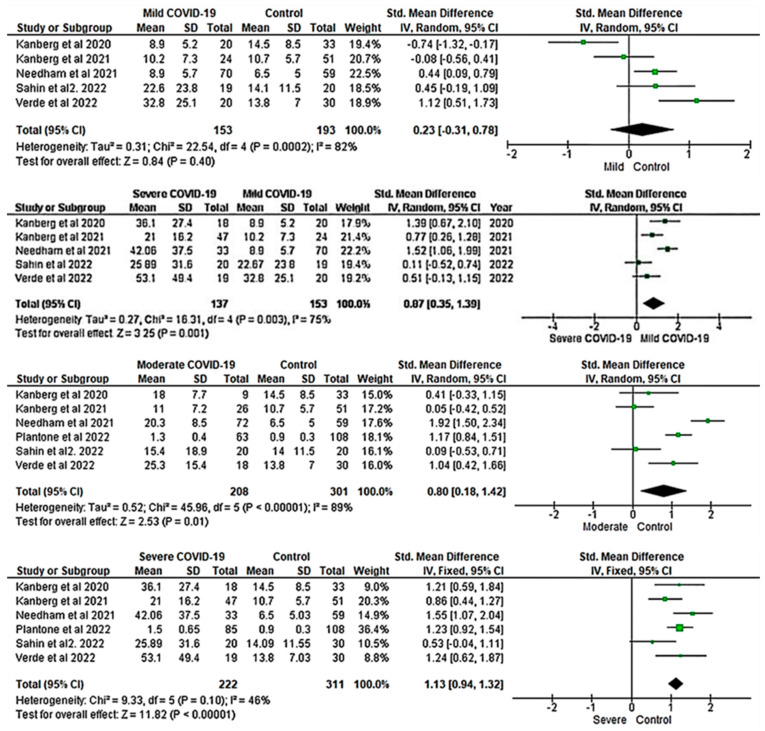
Forest plots show the pooled NfL between COVID-19 severity and healthy controls [17,18,21,25,35,37,59]. Increased blood NfL level is associated with the severity of COVID-19. A positive SMD value represents that the estimated value is significantly higher in COVID-19 patients than in healthy controls. The y-axis represents included studies, and the x-axis of the forest plot represents the effect sizes of the estimate (SMD). The square box (green) in the forest plot represents the SMD (effect estimate) of each study, and the area of the square box indicates the weight assigned to each study. The length of the horizontal line running across each study represents the width of the 95% CI for the SMD estimate for each study. The neutral point is plotted on the x-axis at the “0” mark. A vertical line passes through the neutral point and indicates the study groups (COVID-19 vs. Control). The black diamond represents the overall SMD estimate in the form of the inverse variance random- or fixed-effects meta-analysis. CI, confidence interval; COVID-19, Coronavirus disease 2019; IV, inverse variance; SD, standard deviation; Std, standard.

Five studies were included to investigate the association between NfL and COVID-19 severity. RE meta-analysis of the overall pooled NfL revealed no significant difference between patients with mild COVID-19 and healthy controls (SMD = 0.23; 95% CI: −0.31, 0.78; *p* > 0.05; I^2^: 82%) (first panel from the top, Figure 5). However, there was a significant decrease in NfL levels in severe COVID-19 patients when compared to mild cases (SMD = 0.87; 95% CI: 0.35, 1.39; Z = 3.25; *p* ≤ 0.001; I^2^: 75%) (second panel from the top, Figure 5). 

Six studies were included in the meta-analysis of pooled NfL levels between patients with moderate and severe COVID-19 and healthy controls. The RE meta-analysis demonstrated that NfL levels in patients with moderate COVID-19 were significantly higher than in healthy controls (SMD = 0.80; 95% CI: 0.18, 1.42; *p* ≤ 0.01), with significant heterogeneity (I^2^: 89%; *p* ≤ 0.001) (third panel from the top, Figure 5). The pooled fixed-effects meta-analysis revealed a significant difference in NfL levels between patients with severe COVID-19 and healthy controls (SMD = 1.13; 95% CI: 0.94, 1.32; Z = 11.82; *p* ≤ 0.001), although there was no significant heterogeneity (I^2^: 46%; *p* > 0.05) (bottom panel, Figure 5).

#### 3.2.5. Low Risk of Publication Bias Observed in GFAP and NfL Studies

**Figure 6:**
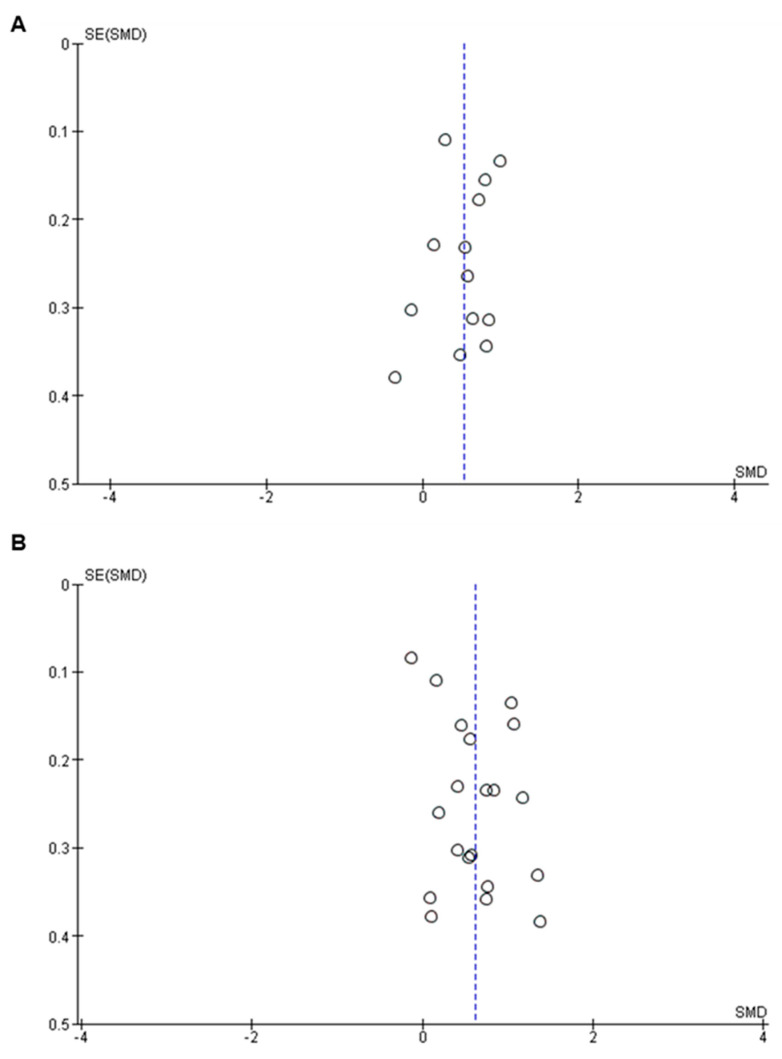
Funnel plot showing low risk of publication bias in GFAP (**A**) and NfL (**B**) studies. The x-axis of the funnel plot represents the effect estimates (SMD) of the studies, and the y-axis of the funnel plot represents the standard error of SMD. The vertical line (blue) represents the summary estimate (SMD). Each circle symmetrical to the blue line represents individual studies. SE, standard error; SMD, standard mean difference.

In this study, we conducted a thorough analysis of heterogeneity and publication bias within the included studies (Tables S1–S3). Higgin’s I-squared statistics showed that the heterogeneity of the included studies was high for both GFAP (I^2^: 66%; *p* ≤ 0.001) (top panel, Figure 2) and NfL (I^2^: 84%; *p* ≤ 0.001) (top panel, Figure 4). To further assess publication bias, we visually inspected the funnel plot for each analysis. The results showed that there was a low risk of publication bias for both GFAP (Figure 6A) and NfL (Figure 6B) analyses.

#### 3.2.6. Subgroup Analysis Showed Increased GFAP in COVID-19 Patients

**Figure 7:**
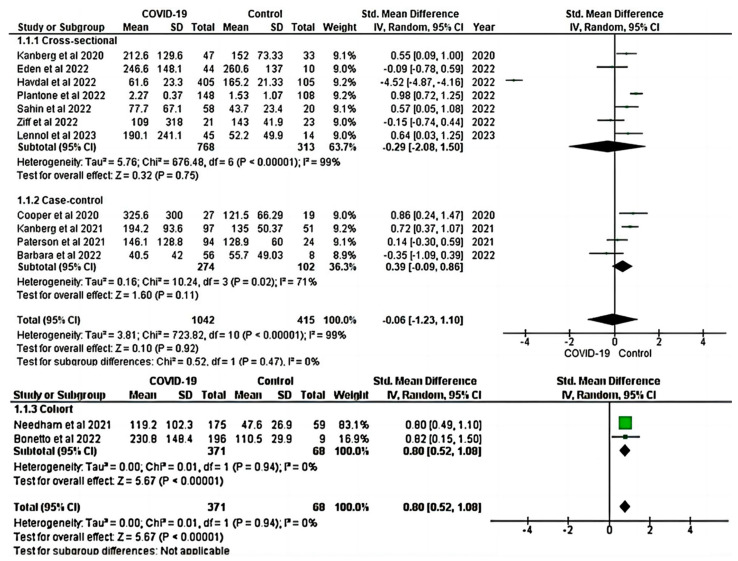
Forest plots of subgroup analysis for the overall pooled SMD of GFAP by study design [17,18,20,35,37,56,57,58,59,60,62,64,65]. Subgroup analysis showed increased GFAP in COVID-19 patients in cohort studies (**bottom panel**) but not in cross-sectional and case–control studies (**top panel**). A positive SMD value represents that the estimated value is significantly higher in COVID-19 patients than in healthy controls. The y-axis represents included studies, and the x-axis represents the effect sizes of the estimate (SMD). The square box in the forest plot represents the SMD (effect estimate) of each study, and the area of the square box (green) indicates the weight assigned to this particular study. The length of the horizontal line running across each study estimate represents the width of the 95% CI for the SMD estimate for that particular study. The vertical neutral point is plotted on the x-axis at the “0” mark. The black diamond represents the overall SMD estimate in the form of the inverse variance random effects meta-analysis. CI, confidence interval; COVID-19, Coronavirus disease 2019; GFAP, glial fibrillary acidic protein; IV, inverse variance; SD, standard deviation; Std, standard.

Subgroup analysis was conducted to explore the overall pooled SMD of GFAP between COVID-19 patients and healthy controls based on the study design. Subgroup analysis by study design showed significant differences among the groups in cohort studies (SMD = 0.80; 95% CI: 0.52, 1.08; *p* ≤ 0.001; I^2^: 0.0%) (bottom panel, Figure 7). However, no significant difference was observed among the groups in cross-sectional and case–control studies ((SMD = −0.29; 95% CI: −2.08, 1.5; *p* > 0.05; I^2^: 99%) and (SMD = 0.39; 95% CI: −0.09, 0.86; *p* > 0.05; I^2^: 71%)), respectively (top panel, Figure 7).

#### 3.2.7. Subgroup Analysis Showed Increased NfL in COVID-19 Patients

**Figure 8:**
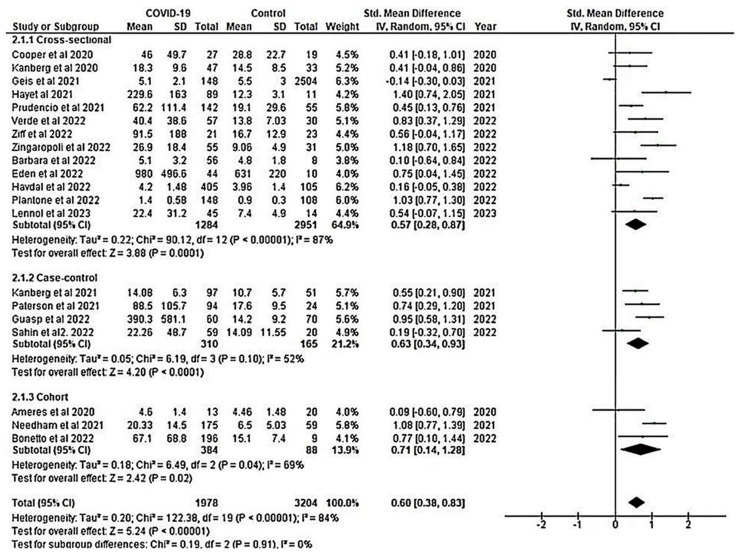
Forest plots of subgroup analysis for the overall pooled SMD of NfL by study design [17,18,19,20,25,26,27,32,35,37,56,57,58,59,60,61,62,63,64,65]. Subgroup analysis showed increased NfL in COVID-19 patients in all three study designs. A positive SMD value represents the estimated value is significantly higher in COVID-19 patients than in healthy controls. The y-axis represents included studies, and the x-axis represents the effect sizes of the estimate (SMD). The square box (green) in the forest plot represents the SMD (effect estimate) of each study, and the area of the square box indicates the weight assigned to this particular study. The length of the horizontal line running across each study estimate represents the width of the 95% CI for the SMD estimate for that particular study. The vertical neutral point is plotted on the x-axis at the “0” mark. The black diamond represents the overall SMD estimate in the form of the inverse variance random-effects meta-analysis). CI, confidence interval; COVID-19, Coronavirus disease 2019; IV, inverse variance; NfL, neurofilament light chain; SD, standard deviation; Std, standard.

Subgroup analysis was conducted on the overall pooled SMD of NfL between COVID-19 and healthy controls based on the study design. The results showed that there was a significant difference in NfL between the two groups in cross-sectional, case–control, and cohort studies with an overall pooled SMD of 0.57 (95% CI: 0.28, 0.87), 0.63 (95% CI: 0.34, 0.93), and 0.71 (95% CI: 0.14, 1.28), respectively (Figure 8).

## 4. Discussion

COVID-19 is a significant public health concern, and it continues to challenge healthcare systems worldwide [66]. While the virus primarily affects the respiratory system, its impact in the presence of the angiotensin-converting enzyme 2 (ACE2) receptor in neuronal cells indicates that it can potentially serve as a route for SARS-CoV-2 to invade the brain. This phenomenon could explain neurological symptoms such as anosmia, dysgeusia, and headaches [67]. A growing body of evidence suggests neurologic involvement during or after acute infection, resulting in various neurological manifestations and complications [19,20,25]. Serum GFAP and NfL have recently been considered as potential biomarkers of several neurological complications and their severity [15,22]. Exploring the relationship between neurological biomarkers (GFAP and NfL) and COVID-19 could be of great clinical value. However, a comprehensive analysis of the relationship between neurological biomarkers and COVID-19 infection is still limited. Therefore, this systematic review and meta-analysis aimed to determine the pooled SMD of GFAP and NfL between COVID-19 patients and healthy controls and to generate evidence for the association between neurological injury-related biomarkers and the severity of COVID-19 infection. 

To determine the association of GFAP and NfL with COVID-19, we pooled the SMD from included studies. The majority of the studies included in our analysis showed significantly elevated GFAP levels in COVID-19 patients compared to healthy controls [17,20,35,37,62,65], while some studies demonstrated contradictory findings [31,53,60]. We included a total of 13 studies comprising 1896 participants in the meta-analysis of the association between GFAP and COVID-19 infection. Our overall pooled results showed that the level of GFAP was significantly higher in patients with COVID-19 than in healthy controls. This finding was consistent with Plantone et al. [18], who found significantly higher serum GFAP levels in patients with COVID-19 compared to the healthy controls. Cooper et al. also reported significantly two-fold higher GFAP levels in critically ill patients with COVID-19 compared to the healthy controls [65]. In addition, serum GFAP levels were increased in patients with severe brain injury on admission and significantly predicted neurological outcomes at six months [22], and elevated levels of serum GFAP were significantly correlated with the extent of brain damage in ischemic stroke patients [15].

Different studies revealed that levels of GFAP significantly increased in COVID-19 patients with fatal outcomes [42,56]. We observed a significant difference in the pooled GFAP levels between survivor and non-survivor groups of COVID-19 patients (bottom panel, Figure 2). This finding was consistent with Frontera et al. [50], who found significantly elevated levels of GFAP in COVID-19 patients who died in the hospital when compared to survivors [50]. Hege et al. also reported significantly higher GFAP concentration in non-survivor COVID-19 patients when compared to survivor groups [41]. In addition, another study concluded that patient age and levels of serum GFAP were significant predictors of in-hospital COVID-19-associated mortality [18].

The pooled level of GFAP was significantly higher in patients with severe COVID-19 compared to mild groups, although there was no significant difference in the pooled GFAP level between mild COVID-19 and healthy controls (Figure 3). However, in this meta-analysis, we observed a significant difference in the overall pooled GFAP level in patients with moderate and severe COVID-19 when compared to the healthy controls (Figure 3). Our finding was consistent with Sahin et al. [21], who found significantly higher GFAP levels in patients with severe COVID-19 than in the healthy controls. Kanberg et al. also reported that patients with moderate and severe COVID-19 had significantly higher concentrations of GFAP than healthy controls [35]. 

The changes in neurological biomarkers observed in COVID-19 patients may be attributed to a combination of direct effects of viral infection or post-infectious inflammation as well as complications arising from prolonged intensive care [8,53]. NfL is a component of the axonal cytoskeleton and is recognized as a marker of neurological injuries in several CNS infections [10,68]. Blood biomarkers associated with neurological injury offer additional information for different injury processes, which can aid in the management of patients, diagnosis, and prognosis for treatment [69,70]. Numerous studies have reported alterations in NfL levels in COVID-19 patients [18,21,25,26,35,62,65]. Therefore, a comprehensive assessment of the association between NfL and COVID-19, mortality, and disease severity may have significant value in identifying COVID-19 patients at high risk and predicting prognostic outcomes.

This systematic review and meta-analysis included 20 studies, comprising a total of 5182 participants, to analyze the association between NfL levels and COVID-19. The pooled meta-analysis revealed significantly higher NfL levels in patients with COVID-19 when compared to the healthy controls (top panel, Figure 4). Similar findings were reported in several studies [18,25,27], indicating that COVID-19 patients had higher NfL levels than healthy controls. In addition, different studies revealed that elevated levels of NfL were significantly associated with fatal outcomes in COVID-19 patients [19,41,42]. Serum NfL levels at the time of hospital admission significantly predicted patients who were at high risk of COVID-19-associated mortality [18]. Our results corroborate these findings as the overall pooled meta-analysis demonstrated a significant difference in NfL levels between survivor and non-survivor groups of COVID-19 patients (bottom panel, Figure 4). This finding was consistent with Frontera et al. [50], who found significantly elevated levels of NfL in COVID-19 patients who died in the hospital when compared to survivor groups [50].

The overall pooled NfL levels did not show significant differences between mild COVID-19 patients and healthy controls (top panel, Figure 5). However, when compared to patients with moderate COVID-19, NfL levels were considerably higher in those with severe cases (second panel from the top, Figure 5). Our results aligned with the report by Kanberg et al. [35], who found that patients with severe COVID-19 had higher concentrations of NfL than those with mild disease. Additionally, the pooled NfL level in patients with moderate COVID-19 was significantly higher than in healthy controls (third panel from the top, Figure 5). Different studies have suggested a significantly higher level of NfL in patients with severe COVID-19 when compared to healthy controls (bottom panel, Figure 5) [17,35]. Similarly, in this meta-analysis, we observed significant differences in the pooled NfL levels between severe COVID-19 and healthy controls. This finding was consistent with Kanberg et al. [35], who found that patients with severe COVID-19 had higher concentrations of NfL than healthy controls. 

NfL is an intra-axonal structural protein critical for structural stability and radial growth of axons. It can be measured in blood as a marker of neuronal injury because it has a low molecular weight (68 kDa) and readily diffuses from parenchyma to blood and CSF upon the neuronal injury [16,28]. This process is part of the body’s response to CNS injury and inflammation. GFAP, a cytoskeletal protein, forms a junction between the nucleus and the cell membrane and is involved in intracellular cytoskeletal reorganization. It is a cell-specific marker highly expressed in astrocytes and engaged in cell communication and functioning of BBB, regulating astrocyte mechanical strength, morphology, and stability [8]. The BBB is formed by capillary endothelial cells and surrounded by specific ends of basal and perivascular astrocytes. Astrocytes play an important role in the maintenance and regulation of the BBB. They help ensure that the environment inside the CNS is stable, limit the entry of harmful substances, and support the normal function of neurons. Therefore, the disruption of BBB can result in leakage of GFAP into the bloodstream. GFAP serves as a biomarker of astrocytic injury because it is released into the blood and CSF during brain injury associated with the increased functional activity of astrocytes or injury [23,27]. 

These biomarkers could be ingested and processed by phagocytes and released from damaged CNS cells into the circulation as intact proteins. They do this through endocytosis and the subsequent delivery of these proteins to lysosomes for degradation. The increased concentrations of NfL and GFAP in the bloodstream relate to damage to the nerve tissue and the development of neurodegenerative states during brain injury [10,24]. This makes GFAP and NfL attractive biomarkers for screening neurologic injury.

The meta-analysis provides insights into the association between neurological biomarkers and COVID-19. It showed that the levels of GFAP and NfL were elevated in COVID-19 patients when compared to healthy controls, indicating the presence of neurological injury. These biomarkers could potentially serve as a useful tool for identifying COVID-19 patients at higher risk of developing neurological complications, allowing for early diagnosis and management. Subgroup analysis by study design revealed that the differences in biomarker levels were consistent across different study types (Figure 7 and Figure 8). These findings have important implications for long COVID-19 monitoring and predicting long-term neurological complications as well as COVID-19-associated mortality.

In this systematic review and meta-analysis, we conducted a thorough analysis of recent research studies and identified the associated biomarkers that could be used for monitoring and evaluating COVID-19 patients with neurological complications. We have performed our study by strictly following standard guidelines such as the PRISMA flow chart. Furthermore, we have assessed the quality of each article using the modified NOS quality assessment tool. However, when interpreting the findings, it is important to take into account the present study’s limitations. One limitation might be the heterogeneity among the included studies, which could have impacted the accuracy and reliability of the findings. The majority of the studies included in this meta-analysis were conducted in Europe, which could potentially introduce regional bias. In addition, the small sample size in some of the included studies may have influenced the statistical power of the analyses.

## 5. Conclusions

The present meta-analysis revealed a significant association between COVID-19 and elevated levels of GFAP and NfL. GFAP and NfL levels were significantly higher in COVID-19 non-survivors than in COVID-19 survivors. Additionally, the pooled GFAP and NfL levels were significantly lower in patients with mild COVID-19 compared to severe cases, while the pooled level of GFAP and NfL were significantly increased in patients with moderate and severe COVID-19 when compared to the healthy controls. These findings suggest that GFAP and NfL may serve as potential neurological biomarkers for the early diagnosis and management of COVID-19 patients.

## Figures and Tables

**Table 1:** Summary of GFAP level between COVID-19 and healthy controls from the pooled SMD analysis.

S.N.	Authors	Year	Study Design	Country	Sample Size of COVID-19	Mean GFAP of COVID-19	SD of GFAP COVID-19	Sample Size of Control	Mean GFAP of Control	SD of GFAP in Control	NOS
1	Cooper et al. [65]	2020	Cross-sectional	Canada	27	325.60	300.00	19	121.50	66.29	8
2	Kanberg et al. [17]	2020	Cross-sectional	Sweden	47	212.60	129.60	33	152.00	73.33	8
3	Kanberg et al. [35]	2021	Case–control	Sweden	97	194.20	93.60	51	135.00	50.37	7
4	Paterson et al. 60]	2021	Case–control	UK	94	146.10	128.80	24	128.90	60.00	7
5	Needham et al. [59]	2021	Cohort	UK	175	119.20	102.30	59	47.60	26.90	7
6	Sahin et al. [21]	2022	Cross-sectional	Turkey	58	77.70	67.10	20	43.70	23.40	7
7	Plantone et al. [18]	2022	Cross-sectional	Italy	148	2.27	0.37	108	1.53	1.07	7
8	Barbara et al. [62]	2022	Cross-sectional	USA	56	40.50	42.00	8	55.70	49.03	7
9	Eden et al. [57]	2022	Cross-sectional	Sweden	44	246.60	148.10	10	260.30	137.00	7
10	Havdal et al. [20]	2022	Cross-sectional	Norway	405	61.60	23.30	105	165.20	21.33	7
11	Bonetto et al. [56]	2022	Cohort	Italy	196	230.80	148.40	9	110.50	29.90	8
12	Ziff et al. [58]	2022	Cross-sectional	UK	21	109.00	318.00	23	143.00	41.90	8
13	Lennol et al. [64]	2023	Cross-sectional	Spain	45	190.10	241.10	14	52.20	49.90	8

COVID-19, Coronavirus disease 2019; GFAP, glial fibrillary acidic protein (pg/mL); NOS, Newcastle–Ottawa scale; SD, standard deviation; S.N., serial number; UK, United Kingdom; USA, United States of America.

**Table 2:** Summary of NfL level between COVID-19 and healthy controls from the pooled SMD analysis.

S.N.	Authors	Year	Study Design	Country	Sample Size of COVID-19	Mean NfL of COVID-19	SD of NfL in COVID-19	Sample Size of Control	Mean NfL of Control	SD of NfL in Control	NOS
1	Cooper et al. [65]	2020	Cross-sectional	Canada	27	46.00	49.70	19	28.80	22.70	8
2	Kanberg et al. [17]	2020	Cross-sectional	Sweden	47	18.30	9.60	33	14.50	8.50	8
3	Ameres et al. [63]	2020	Prospect-cohort	German	13	4.60	1.40	20	4.46	1.48	7
4	Kanberg et al. [35]	2021	Case–control	Sweden	97	14.08	6.30	51	10.70	5.70	7
5	Paterson et al. [60]	2021	Case–control	UK	94	88.50	105.70	24	17.60	9.50	7
6	Geis et al. [32]	2021	Cross-sectional	German	148	5.10	2.10	2504	5.50	3.00	8
7	Prudencio et al. [19]	2021	Cross-sectional	USA	142	62.20	111.40	55	19.10	29.60	8
8	Hay et al. [61]	2021	Cross-sectional	USA	89	229.60	163.00	11	12.30	3.10	7
9	Needham et al. [59]	2021	Cohort	UK	175	20.33	14.50	59	6.50	5.03	7
10	Guasp et al. [26]	2022	Case–control	Spain	60	390.30	581.10	70	14.20	9.20	8
11	Verde et al. [25]	2022	Cross-sectional	Italy	57	40.40	38.60	30	13.80	7.03	7
12	Plantone et al. [18]	2022	Cross-sectional	Italy	148	1.40	0..58	108	0.90	0.30	7
13	Barbara et al. [62]	2022	Cross-sectional	USA	56	5.10	3.20	8	4.80	1.80	7
14	Eden et al. [57]	2022	Cross-sectional	Sweden	44	980.00	496.60	10	631.00	220.00	7
15	Havdal et al. [20]	2022	Cross-sectional	Norway	405	4.20	1.48	105	3.96	1.40	7
16	Bonetto et al. [56]	2022	Cohort	Italy	196	67.10	68.80	9	15.10	7.40	8
17	Ziff et al. [58]	2022	Cross-sectional	UK	21	91.50	188.00	23	16.70	12.90	8
18	Zingaropoli et al. [27]	2022	Cross-sectional	Italy	55	26.90	18.40	31	9.06	4.90	8
19	Sahin et al. [37]	2022	Case–control	Turkey	59	22.26	48.70	20	14.09	11.55	7
20	Lennol et al. [64]	2023	Cross-sectional	Spain	45	22.40	31.20	14	7.40	4.90	8

COVID-19, Coronavirus disease 2019; NfL, neurofilament light chain (pg/mL); NOS, Newcastle–Ottawa scale; SD, standard deviation; S.N., serial number; UK, United Kingdom; USA, United States of America.

## Data Availability

Not applicable.

## Supplementary Materials

The following supporting information can be downloaded at https://www.mdpi.com/article/10.3390/ijms242115738/s1.

## Abbreviations

AD	Alzheimer’s disease
BBB	Blood–brain barrier
CI	Confidence interval
CNS	Central nervous system
COVID-19	Coronavirus disease 2019
CSF	Cerebrospinal fluid
FiO_2_	Fractional inspired oxygen
GFAP	Glial fibrillary acidic protein
ICU	Intensive care unit
IQR	Interquartile range
NfL	Neurofilament light chain
NOS	Newcastle–Ottawa scale
PaO_2_	Partial pressure of oxygen
PRISMA	Preferred reporting items for systematic reviews and meta-analysis
RE	Random effect
SARS-CoV-2	Severe acute respiratory syndrome coronavirus-2
SD	Standard deviation
SMD	Standardized mean differences
UK	United Kingdom
USA	United States of America
