# Supplementary material for: Blood Biomarkers as Prognostic Indicators for Neurological Injury in COVID-19 Patients: A Systematic Review and Meta-Analysis
Source: Int J Mol Sci. 2023 Oct 30;24(21):15738. doi: 10.3390/ijms242115738 (PMC10649265; doi:10.3390/ijms242115738)
Supplement: Supplementary file 1 [file ijms-24-15738-s001.zip › ijms-2642132-supplementary.pdf]

Supplementary Materials

Table S1. Risk of bias assessment for cross-sectional studies by the Newcastle-Ottawa-Scale.

**Authors***	**Selection**				**Comparability**	**Outcome**		**NOS** score**	**Risk of bias**
	Representativeness	Sample size	Non-response rate	Ascertainment of the screening tool		Assessment of outcome	Statistical test		
Sahin et al. 2022 [21]	1	0	0	2	1	2	1	7	Low
Plantone et al. 2022 [18]	1	0	0	2	1	2	1	7	Low
Kanberg et al. 2020 [17]	1	0	1	2	1	2	1	8	Low
Eden et al. 2022 [57]	1	0	0	2	1	2	1	7	Low
Havdal et al. 2022 [20]	1	0	1	2	1	2	1	8	Low
Lennol et al. 2023 [64]	1	0	1	2	1	2	1	8	Low
Ziff et al. 2022 [58]	1	0	1	2	1	2	1	8	Low
Zingaropoli et al. 2022 [27]	1	0	1	2	1	2	1	8	Low
Verde et al. 2022 [25]	1	0	1	2	0	2	1	7	Low
Geis et al. 2021 [32]	1	1	1	2	1	2	1	8	Low
Prudencio et al. 2021 [19]	1	0	1	2	1	2	1	8	Low
Hay et al. 2021 [61]	1	0	0	2	1	2	1	7	Low
Cooper et al. 2020 [65]	1	0	1	2	1	2	1	8	Low
Barbara et al. 2022 [62]	1	0	1	2	0	2	1	7	Low

Table S2. Risk of bias assessment for cohort studies by the Newcastle-Ottawa-Scale.

**Authors***	**Selection**				**Comparability**	**Outcome**			**NOS score**	**Risk of bias**
	Representativeness	Selection of the non-exposed	Ascertainment of exposure	outcome of interest was not present at the start of study		Assessment of outcome	Time of follow-up	Adequacy of follow up		
Bonetto et al. 2022 [56]	1	1	1	1	1	2	0	1	8	Low
Needham et al. 2021 [59]	1	0	1	1	1	1	1	1	7	Low
Ameres et al. 2020 [63]	1	0	1	1	1	1	1	1	7	Low

Table S3. Risk of bias assessment for case-control studies by the Newcastle-Ottawa-Scale.

**Authors ***	**Selection**				**Comparability**	**Exposure**			**NOS score**	**Risk of bias**
	Is the case definition adequate	Representativeness of the cases	Selection of Controls	Definition of Controls		Ascertainment of exposure	Same method of ascertainment for cases and controls	Non-Response rate		
Kanberg et al 2021. [35]	1	1	0	1	1	1	1	1	7	Low
Paterson et al. 2021 [60]	1	1	0	0	1	2	1	1	7	Low
Guasp et al. 2022 [26]	1	1	0	1	1	2	1	1	8	Low
Sahin et al,2022 [37]	1	1	0	0	2	1	1	1	7	Low

*The reference lists are available in the main text. ** Newcastle-Ottawa-Scale

Note: Studies that received a total score of 8 or 7 points were categorized as having a low risk of bias or high quality. Those scoring 6 points were considered to have a medium risk of bias or moderate quality, while studies with a score of 5 points or less were regarded as having a high risk of bias or low quality. In terms of selection, studies were assessed as having a low, medium, or high risk of bias based on scores of 3, 1-2, or 0 points, respectively. For comparability, studies were evaluated as having a low, medium, or high risk of bias depending

on scores of 2, 1, or 0 points, respectively. Regarding outcome, studies were categorized as having a low, medium, or high risk of bias based on scores of 3, 2, or 1 point, respectively.
